# Phenotypic and Cytogenetic Characterization of Mesenchymal Stromal Cells in *De Novo* Myelodysplastic Syndromes

**DOI:** 10.1155/2016/8012716

**Published:** 2016-08-29

**Authors:** A. J. I. S. Rathnayake, H. W. W. Goonasekera, V. H. W. Dissanayake

**Affiliations:** ^1^Human Genetics Unit, Faculty of Medicine, University of Colombo, 00800 Colombo, Sri Lanka; ^2^Department of Pre-Clinical Sciences, Faculty of Medicine, General Sir John Kotelawala Defence University, Ratmalana, Sri Lanka

## Abstract

Bone marrow (BM) mesenchymal stem/stromal cells (MSCs) are vital in hematopoiesis. Whether BM-MSCs alter their characteristics in Myelodysplastic Syndromes (MDS) is still controversial. We characterized MSCs of* de novo* MDS patients in Sri Lanka who have not been reported previously in the literature. We also analyzed MSCs derived from different MDS subtypes. MSCs were culture-expanded, characterized by flow cytometry, and induced towards osteogenic and adipogenic differentiation. Growth properties were determined using growth curves and population doubling times. Karyotyping and FISH were performed on MSCs. Cell morphology, differentiation potential, and CD marker expression of MDS-MSCs of all subtypes were comparable to those of control-MSCs. No significant growth differences were observed between control MSCs and MDS-MSCs of all subtypes (*p* > 0.05). 31% of MDS-MSCs had chromosomal aberrations (der(3),del(6q),del(7p), loss of chromosomes) whose BM karyotypes were normal. Highest percentage of karyotypic abnormalities was observed in RCMD-MSCs. Patients with abnormal BM karyotypes had no aberrant MSC clones. Results show that in spite of presence of genetically abnormal clones in MDS-MSC populations,* in vitro* phenotypic and growth characteristics of MSCs in MDS remain unchanged. Further, the occurrence of genetic abnormalities in BM-MSCs in MDS could be considered as an autonomous event from that of their hematopoietic counterparts.

## 1. Introduction

Multipotent mesenchymal stem/stromal cells (MSCs) are a group of undifferentiated cells, with the ability of self-renewal and differentiation into multiple cell lineages including adipocytes, osteocytes, and chondrocytes [1, 2]. They are characterized by the expression of CD73, CD90, and CD105 and lack expression of hematopoietic markers such as CD34 and CD45 [1]. Bone marrow (BM) resident MSCs regulate hematopoiesis, homeostasis, and maintenance of hematopoietic stem cells (HSCs) through direct cell to cell contacts and by secretion of regulatory factors [3, 4]. Biological characteristics of MSCs, the most vital component in the functioning niche for HSCs, are thought to be altered in hematological malignancies [5–8].

Myelodysplastic Syndromes (MDS) are a complex bone marrow hematopoietic stem cell disorders characterized by peripheral cytopenia, cellular dysplasia, and dysfunction resulting in an ineffective hematopoiesis [6, 7]. Emerging research and* in vitro* models demonstrate that the disease is not simply derived from an abnormal HSC clone but also is a combined result of defective cells in the marrow microenvironment and their complex interactions with hematopoietic compartment [6, 7]. Functional integrity of BM-MSCs in MDS and their contribution in pathogenesis and progression of MDS are controversial [5–12]. While some reports have found that the stromal compartment in MDS is cytogenetically and functionally normal [10, 11], other researches have shown that the MSCs are cytogenetically abnormal and defective in carrying out their normal functions [5, 12, 13]. The groups which claim for the view of MDS-MSCs are normal, have shown that the cellular morphology, expression of surface antigens, and differentiation ability of MDS-MSCs are comparable to normal MSCs, and are devoid of chromosomal aberrations [10, 14, 15]. In contrast, some other studies have reported altered proliferative and differentiation capacities, immunomodulatory properties, cell-cell interactions, signaling pathways, and cytogenetic profiles [13, 16–20]. Coexistence of genetic aberrations in both hematopoietic and mesenchymal stem cell compartments was reported previously [12, 21–23] and the aberrations in MSCs are found to be present more frequently in patients with cytogenetically abnormal HSCs [21]. Whether the abnormalities occur in both compartments together with a clonal relationship or in an independent manner is still not clear [21–23]. Therefore it is essential to carry out further research on their phenotypic and cytogenetic properties to address the existing controversies and to better understand the disease biology.

The cytogenetic characteristics of BM of MDS have shown differences in Asian populations compared to the Western people; +8 and del(7q) are the most frequent cytogenetic abnormality in Asian MDS patients whereas del(5q) has been reported as the most common cytogenetic abnormality in Western MDS patients [24–29]. It is also reported that the age of onset in Asia is younger than in Western countries [28, 29]. Most of the studies have been done using North American and European populations and data on South Asian MDS patient populations are sparse. Therefore, although the biological features and cytogenetic profiles of MSCs have been reported previously [9, 12, 21–23] it is important to further explore the phenotypic and cytogenetic features of MDS patients in different ethnicities due to population differences in cell profiles [29, 30]. Further, the studies which compare the characteristics of MSCs derived from different MDS subtypes are limited. Hence, this study was carried out on Sri Lankan primary MDS patients who have not been reported previously in the literature in order to better understand the characteristics of MDS-MSCs as well as fill the deficiency of data from South Asian context.

In this study we analyzed the morphology, immunophenotypes, differentiation ability, growth, and cytogenetic profiles of the BM-MSCs from a newly diagnosed* de novo* MDS patient group in Sri Lanka. We also compared the characteristics of MSCs derived from different subtypes of MDS.

## 2. Materials and Methods

### 2.1. Patients

Twenty patients diagnosed as having* de novo* MDS were recruited from 3 tertiary care general hospitals (National Hospital of Sri Lanka, Colombo South Teaching Hospital, and Colombo North Teaching Hospital) and a cancer care hospital (National Cancer Institute, Maharagama) in Sri Lanka. Patients reported to above hospitals from 2013 to 2015 were recruited. MDS diagnosis was based on clinical presentation, peripheral blood counts, BM aspiration, trephine biopsy reports, and BM karyotypes and classified into subtypes according to the WHO classification of 2008. Patients with secondary MDS and other chronic diseases were excluded. Five persons who have an indication to undergo bone marrow aspiration as part of their diagnostic work-up other than a primary/secondary bone marrow failure syndrome were also recruited for the study as controls. The study was carried out according to the Declaration of Helsinki (2008). The protocol was approved by the Ethics Review Committee of the Faculty of Medicine, University of Colombo (ERC-12-40), and all necessary permits were obtained from the abovementioned hospitals to recruit their patients for the described study. Bone marrow samples for the study were obtained at the time of initial diagnostic BM aspiration under local anesthesia. An information sheet which contained details of purpose of the study, duration, procedures, confidentiality, potential risks, hazards, discomforts, and so forth and a consent form were prepared in all the three national languages used in Sri Lanka. The information sheet was given to the patients before the marrow sampling and its content was explained to the patients and their relatives. It was also explained that their participation is voluntary, that they will not be identified in the reporting of the findings, and that they may withdraw at any point before the results of the study are published with no penalty or effect on medical care or loss of benefits. The participants were invited to ask questions before getting the written consent. Written informed consent for the study was obtained prior to BM sample collection.

### 2.2. Isolation and Expansion of MSCs

BM samples (5–10 mL) were collected into heparinized tubes at the time of bone marrow sampling for initial diagnosis of MDS. Isolation of BM MSCs was done using adhesion protocol developed by Colter et al. [31]. The mononuclear cells (MNCs) were isolated from bone marrow using Ficoll gradient. Isolated cells were cultured in T-25 flasks (Corning) at the density of 10^6^ cells/mL in DMEM-KO (knockout) (Gibco) supplemented with 10% FBS (Gibco), 2 mM glutamine (Gibco), and 1% antibiotics (Penicillin-Streptomycin, Gibco) (MSC complete media) and incubated at 37°C in humidified atmosphere of 5% CO_2_ overnight. After 4 days, nonadherent cells with spent cell culture media were discarded from the culture flask, washed with PBS, and were subjected to total medium changes until the appearance of 80% confluent adherent cell layer. The morphology of MSCs was examined daily under an inverted microscope. To detach the cells from the bottom of the flask, cells were incubated with prewarmed 0.05% Trypsin-0.53 mM EDTA and the cells were observed under the inverted microscope for detachment every 30 seconds. When 90% of the cells were detached complete culture media were added to neutralize trypsin, cells were centrifuged, and the pellet was resuspended in prewarmed complete growth medium. An aliquot was taken to determine the total number of viable cells and the cells were subcultured in T75 at a density of 2 × 10^6^ cells/cm^2^. Isolated mesenchymal cells generated from* in vitro* cultures were cryopreserved for future analysis. Same procedures were followed to isolate MSCs from controls.

### 2.3. Morphology and Differentiation Assays

The morphology of cultured MSCs was observed every 3 days under a phase contrast microscope. Morphology was compared with the MSCs isolated from BM of controls.

Differentiation studies were performed at passages 2–4 MSCs. Cells were grown in mesenchymal complete media until they reached 100% confluency. To induce osteogenic differentiation, osteogenic differentiation media (DMEM-HG, 10% FBS, 100 nM dexamethasone, 10 mM *β*-glycerophosphate, and 0.2 mM ascorbate) were added and incubated for 21 days. Media were changed every 3 days. Osteogenic differentiation was detected by Alizarin S stain for calcium deposition. Cells were fixed with 10% formalin (Sigma-Aldrich) for 15 minutes, washed with distilled water, and stained with Alizarin Red S for 45 minutes.

To induce adipogenic differentiation, cells were incubated with adipogenic media (DMED-HG, 1 mM dexamethasone, 0.5 mM 3-isobutyl-1-methyl-xanthine, 10 *μ*g/mL recombinant human insulin, 100 mM indomethacin, and 10% FBS) for 15 days. Adipogenic differentiation was confirmed by the observation of neutral lipid-vacuoles. Cells were fixed with 10% formalin and stained with Oil Red O (Sigma-Aldrich) for 15 minutes.

### 2.4. Immunophenotype Analysis of MSCs after Expansion

Passage 2 MSCs obtained from MDS patients and controls were trypsinized and analyzed by flow cytometry using CD34-phycoerythrin (PE), CD73-fluorescein isothiocyanate (FITC), CD90-PE, CD105-FITC, and CD45-FITC (BD, USA). 10,000 labeled cells were acquired and analyzed using a FACScalibur flow cytometer (BD, USA). The antibody combinations were used as follows: CD34-PE and CD73-FITC, CD90-PE and CD45-FITC, and CD105-FITC. Expression of positive CD markers was analyzed according to the MDS subtype, RCUD (*n* = 6), RCMD (*n* = 4), RAEB (*n* = 3), and controls (*n* = 3).

### 2.5. Proliferation Pattern of MDS-MSCs

#### 2.5.1. Growth Curves and Population Doubling Times

MDS and control MSCs of passage 3 at logarithmic growth phase were seeded in 12-well plates at 0.5 × 10^4^ cells/cm^2^ in MSC complete media and grown for 14 days. Duplicate cultures from each sample and control were harvested every 2 days at a regular time for 14 days and viable cell counts were measured by trypan blue exclusion method using a hemocytometer. Growth curves were drawn for control MSCs (*n* = 4) and MDS-MSCs (*n* = 16) and for each subtype (RCUD; *n* = 7, RCMD; *n* = 5, RAEB; *n* = 3 and del(5q); *n* = 1). For each sample cells in duplicate wells were harvested every 2 days and the average was taken. Each data point in the curve represents the mean of each group. For del(5q) (*n* = 1) each data point was drawn taking the mean cell number of two independent wells.

Doubling time was calculated as follows [32]:

(1) $$\begin{array}{c} PDT=\frac{\left(t-t_{0}\right)\mathrm{lg}2}{\mathrm{lg}N_{t}-\mathrm{lg}N_{0}}, \end{array}$$

where *t*_0_ is starting time of the culture, *t* is termination time of the culture, *N*_0_ is initial cell number of the culture, and *N*_*t*_ is ultimate cell number of the culture.

#### 2.5.2. CFU-F Assays

CFU-F assays were performed as described previously [33]. Passage 2 MSCs of MDS patients and controls (100 cells/dish) were plated on 35 mm tissue culture-treated dishes in triplicate in complete MSC medium with antibiotics and cultured for 14 days. Medium was changed every 3 days. At the end of 14 days, colonies were washed with PBS, fixed with methanol, and stained with Wright Giemsa. Clones of >50 cells were scored as CFU-F.

### 2.6. Karyotyping and Fluorescence* In Situ* Hybridization (FISH) Assays

G-banding technique was used to karyotype culture-expanded MSCs. MSCs of passage 3 were seeded at 1 × 10^6^ cells/cm^2^ in RPMI media supplemented with 10% FBS and 2 mM glutamine and grown until they are 70–80% confluent. Two independent cultures were set. Colcemid® (10 mg/mL) was added to each flask to a final dilution of 0.05 *μ*g/mL and then incubated at 37°C overnight (12–14 hrs). Duration of incubation time with colcemid was optimized for our laboratory conditions. To optimize the conditions cultures were incubated for 2 hours and 5 hours and overnight (12–14 hrs) after addition of colcemid and cells were prepared for cytogenetic analysis. Cultures with overnight colcemid incubation yielded the highest number of analyzable metaphases and were considered as the optimum for MSCs in our laboratory conditions. Cells were harvested with 0.05% Trypsin-EDTA, incubated at 37°C with 0.075 M KCl for 30 min, and fixed with freshly prepared fixative (methanol : acetic acid 3 : 1 v/v). Chromosomal spreads for analysis were prepared by dropping the cell suspension on slides at 76–82% humidity and GTL banding was performed. At least 20 metaphases were analyzed.

Karyotypes were given according to the 2013 International System for Human Cytogenetic Nomenclature (ISCN) guidelines [34]. Presence of an abnormal clone was ascertained by the observation of more than two metaphase spreads having the same numerical or structural abnormality. For gain of chromosomes and loss of chromosomes it was ≥2 and ≥3 metaphase spreads, respectively.

FISH was carried out on BM-MSCs of confirmed del(5q) patients using the probe XL 5q31.2/5q33 (D-5042-100-OG, Meta Systems, USA) according to the manufacture's protocol. At least 200 cells were counted by two individuals independently and ≥95% were set as the cut-off.

### 2.7. Statistical Analysis

Standard statistical software was used for statistical analysis. The* t*-test or nonparametric Mann-Whitney* U* test was used for numerical comparison.

## 3. Results

### 3.1. Demographics and Diagnosis

The age of the MDS patients ranged from 31 to 75 years with the median age of 64.5 years. Majority (65%) was women and male to female ratio of MDS patients was 7 : 13. The study group consisted of 11 (55%) patients with refractory cytopenia with unilineage dysplasia (RCUD), 3 (15%) patients with refractory anemia with excess blasts (RAEB), 5 (25%) patients with refractory cytopenia with multilineage dysplasia (RCMD), and 1 (5%) patient with MDS associated with isolated del(5q) (Table 1).

### 3.2. Morphology and Differentiation Potential of MDS-MSCs

**Figure 1:**
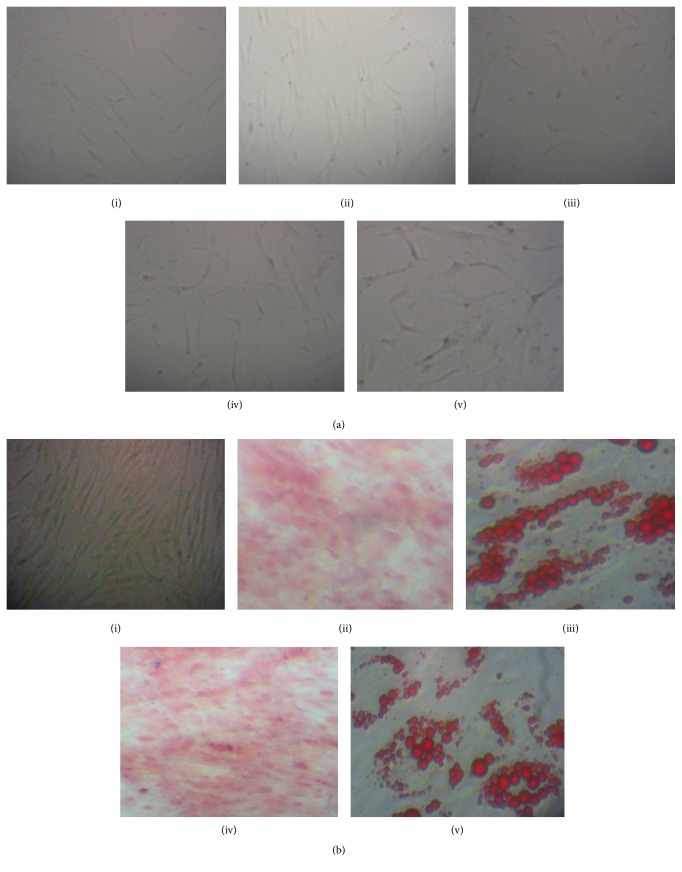
Morphology and differentiation ability of MSCs isolated from controls and MDS patients. (a) Morphology of control and MDS-MSCs. P3 cells of (i) control, (ii) RCUD, (iii) RCMD, (iv) RAEB, and (v) MDS del(5q) at day 4 in culture (20x). (b) (i) Undifferentiated P3 MSCs. (ii) and (iv) are alizarin red stained osteoblasts of control and a MDS patient (14-080) (4x). (iii) and (v) are differentiated control MSCs and MDS-MSCs towards adipogenic cells (40x) as stained with Oil Red O.

Culture-expanded MDS-MSCs of all MDS subtypes and controls showed fibroblast-like, thin spindle-shaped cell morphology (Figure 1(a)). Both control and MDS-MSCs demonstrated adipogenic differentiation as confirmed by the formation of lipid vacuoles stained with Oil Red O after 15 days. Lipid vacuole formation was observed from day 5 onwards after the addition of adipogenic media. All MSCs showed osteogenic differentiation after 21 days as confirmed by staining with alizarin S for calcium deposits (Figure 1(b)). MSCs isolated from all subtypes of MDS patients were capable of differentiating into adipogenic and osteogenic differentiation cell lineages.

### 3.3. MSC Immunophenotype after Expansion

**Figure 2:**
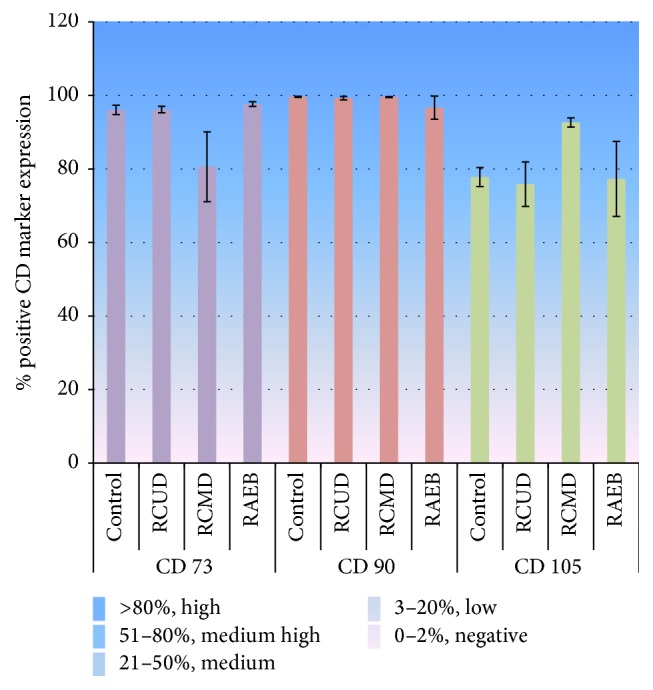
Expression of positive CD markers of culture-expanded P2 MSCs analyzed by flow cytometry. Results were analyzed according to the subtypes: RCUD (*n* = 6), RCMD (*n* = 4), RAEB (*n* = 3), and controls (*n* = 3). Bar graphs represent the mean % of positive cells ± standard error. The expression profile was categorized as follows: negative: 0–2%, low: 3–20%, medium 21–50%, medium high: 51–80%, and high: >80%. All MSCs isolated from controls and MDS subtypes showed high positivity for CD90. Control: 99.6 ± 0.2%, RCUD: 99.3 ± 0.5%, RCMD: 99.6 ± 0.1%, and RAEB: 96.7 ± 3.2%. CD73 expression in RCMD was low (80.6 ± 9.5%) compared to that of control, RCUD, and RAEB patients (96.1 ± 1.3%, 96.2 ± 0.9%, and 97.7 ± 0.6%, resp.). CD105 expression was high in RCMD (>92.6 ± 1.2%) compared to that of control (77.8 ± 2.5%) and RCUD (75.9 ± 6.1%) and RAEB (77.3 ± 10.2%).

MSCs of all patients and controls were positive for CD90 (≥95%), CD73 (≥80%), and CD105 (≥75%) and were negative for CD34 and CD45 (<2%) (supplementary Figures 1 and 2 (in Supplementary Material available online at http://dx.doi.org/10.1155/2016/8012716) for representative FACs plots). Expression of positive markers was analyzed according to the MDS subtype and the expression profile for each subtype was categorized as follows: negative: 0–2%, low: 3–20%, medium 21–50%, medium high: 51–80%, and high: >80% (Figure 2). All MSCs isolated from controls and MDS subtypes showed high positivity for CD90 with >95% mean percentage of positive cells (control: 99.6 ± 0.2%, RCUD: 99.3 ± 0.5%, RCMD: 99.6 ± 0.1%, RAEB: 96.7 ± 3.2%) and high fluorescent intensity (4th logarithmic decade) (supplementary Figure 2). For CD73, mean expression frequencies were control 96.1 ± 1.3%, RCMD 80.6 ± 9.5%, RCUD 96.2 ± 0.9%, and RAEB 97.7 ± 0.6%. In RCMD the percentage of positive cells was low compared to the other subtypes and showed variations in expression levels of CD73 in terms of intensity (10^1^–10^4^ intensity units). CD105 expression was high in RCMD (>92.6 ± 1.2%) compared to that of control (77.8 ± 2.5%), RCUD (75.9 ± 6.1%), and RAEB (77.3 ± 10.2%) (Figure 2).

### 3.4. Proliferation Pattern of MDS-MSCs

**Figure 3:**
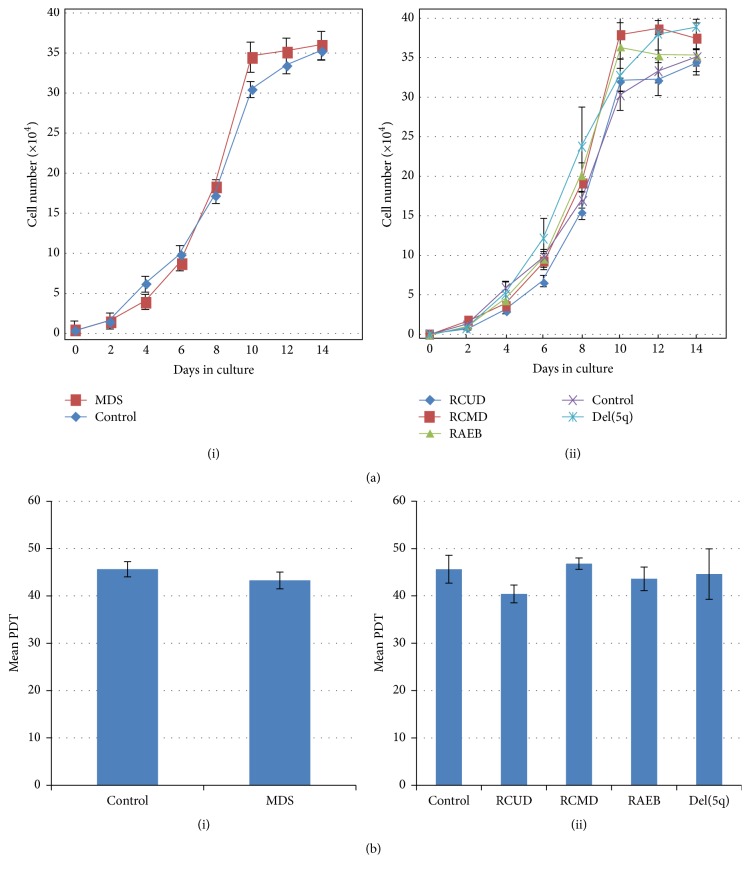
Proliferation pattern of MDS-MSCs. (a) Growth curves of mesenchymal stem cells isolated from MDS patients and control MSCs. (i) Comparison between control MSCs (*n* = 4) and MDS-MSCs (*n* = 16). (ii) Comparison of growth between RCUD (*n* = 7), RCMD (*n* = 5), RAEB (*n* = 3), del(5q) (*n* = 1), and control MSCs. For each sample, cells in duplicate wells were harvested every 2 days at a regular time and the average was taken. Each data point represents the mean of each group ± SEM. For del(5q) (*n* = 1), each data point represents the mean cell number of two independent wells ± SE. (b) Mean population doubling time (PDT). (i) Mean PDT ± SE of control and MDS-MSCs. (ii) Mean PDT ± SE of control, RCUD, RCMD, RAEB, and del(5q). Mean PDT values were not significant between control and MDS-MSCs as well as among the patient subgroups (*p* > 0.05).

Growth curves of MDS-MSCs and control MSCs showed a lag period at 1-2 days of culture and reached an exponential growth at 2–10 days followed by a stationary phase at 10–14 days (Figure 3(a)). Population doubling times (PDT) were 45.50 ± 2.94 hours (h) for controls and 43.15 ± 1.77 h for MDS-MSCs (*p* = 0.48). PDTs for each subtype were RCUD 40.29 ± 1.88 h, RCMD 46.68 ± 1.20 h, RAEB 43.47 ± 2.48 h, and del(5q) 44.43 ± 5.32 h (Figure 3(b)). The PDTs obtained for MDS subtypes were not significantly different from PDT obtained for controls (*p* > 0.05). Mean CFU-F frequency of MDS-MSCs was 19.63 ± 4.91/100 cells (range: 7–41; *n* = 16) and that of controls was 17.93 ± 4.25/100 cells (range: 8–35; *n* = 5) (supplementary Figure 3). CFU-F frequencies of MDS-MSCs were not significantly different from that of control MSCs (*p* > 0.05).

### 3.5. Cytogenetic Analysis of Cultured MDS-MSCs

Normal karyotypes were present in 63% (11/16) of the patient MSCs. 31% (5/16) of the patient MSCs showed abnormal karyotypes (Table 1) including random loss of chromosomes. Three of 5 RCMD patients (60%), 1/6 (16.7%) of RCUD, and 1/3 (33.3%) of RAEB patients had these abnormalities in their MSCs. Of 16 samples karyotyped one culture of MSCs from RCUD patient failed to yield any analyzable metaphase spreads.

**Figure 4:**
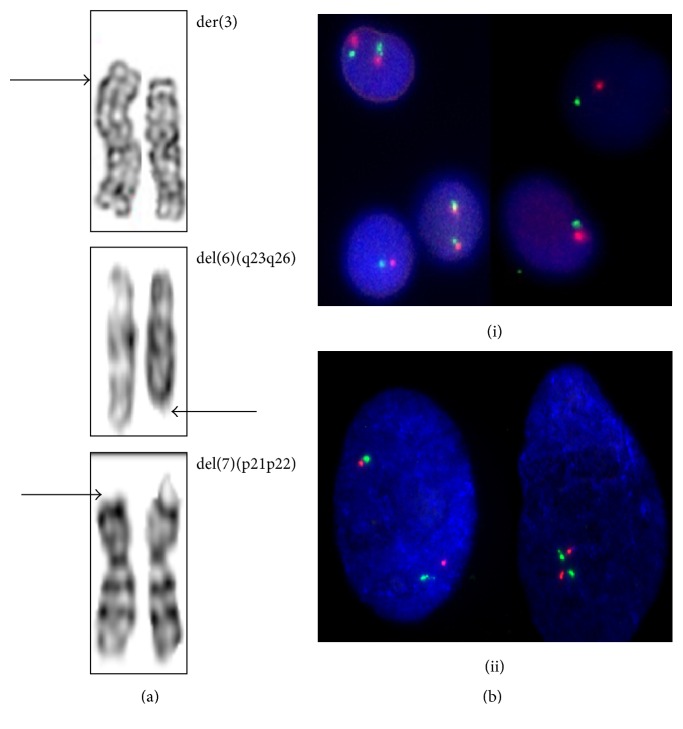
Cytogenetic findings of MDS derived MSCs. (a) Chromosomal abnormalities of MDS-MSCs. Distinct chromosomal abnormalities were found in three patients: 46,XX,der(3) in 14-020, 46,XY,del(6)(q23q26) in 14-072, and 38~43,XY,del(7)(p21p22) in 14-080. (b) FISH analysis of bone marrow (i) and MSCs (ii) of del(5q) patient (14-081). Bone marrow contained two clones; the normal cells showed two red and two green signals and the cells in the abnormal clone showed one red and one green signal. MSCs of this patient showed two red and two green signals indicating the absence of del(5)(q31.2q33).

The aberrations were seen in chromosomes 3, 6, and 7 (Figure 4(a)). Two RCMD and one RAEB patient showed structural abnormalities. One patient diagnosed with RCMD was found to have a derivative of chromosome three with the karyotype, 46,XX,der(3) in eight spreads. MSCs of another patient with RCMD was found to have deletion in the short arm of the chromosome 7 (38~43,XY,del(7)(p21p22)) in addition to the observation of randomly missing chromosomes in most of the metaphase spreads. The RAEB patient with abnormal karyotype had 46,XY,del(6)(q23q26). These patients did not show chromosomal aberrations in their BM karyotypes. The four MDS patients with abnormal BM karyotypes did not show same or any structural abnormality in their MSCs except random loss of chromosomes in one patient (15-017). The karyotype of MSCs of the del 5q patient (whose BM karyotype and FISH were positive for deletion 5q) was normal (46,XX) and the finding was confirmed by FISH analysis of MSCs with two red and two green signals (Figure 4(b)). Loss of chromosome material was observed in most of the MSC spreads. Culture-expanded MSCs of controls showed normal karyotypes.

## 4. Discussion

BM-MSCs are key components in the bone marrow microenvironment which regulate functioning niche of hematopoietic stem cells through direct cellular interactions and by secretion of regulatory factors [2–4]. Some researches have shown that the stromal cell layers derived from MDS marrow are normal while the others declare on their inability to maintain the normal hematopoiesis [10–16, 35–38]. Further, variations in the disease biology and genetic profiles among different ethnicities have been reported [24–28]. Hence it is important to further investigate whether the MSCs in MDS marrow are normal or not. Studying biological characteristics of MDS-MSCs obtained from different populations certainly expand our current knowledge on complex marrow stroma and its role in pathophysiology of MDS. Therefore, with the primary aim of elucidating phenotypic and cytogenetic characteristics of BM-MSCs derived from MDS and to compare these characteristics of MSCs of different MDS subtypes we carried out this study on a group of newly diagnosed* de novo* MDS patients. This study was done on a South Asian MDS group, Sri Lankan primary MDS patients who have not been reported previously in the literature. Hence the study contributes to the global understanding of MSCs by providing data on yet unreported population.

Sixty-nine MDS patients have been reported in Sri Lanka in the year of 2007 according to the latest publication of National Cancer Registry published by the Ministry of Health, Sri Lanka [39]. The study was done on a representative sample (*n* = 20) of Sri Lankan* de novo* MDS patients reported to four main hospitals in Colombo area during the period of 2013–2015.

Isolated MDS-MSCs and control MSCs were assessed for characteristics of MSCs as defined by the International Society for Cellular Therapy (ISCT) [1]. Culture-expanded MDS-MSCs showed plastic adherence and fibroblast like spindle shaped cell morphology. In keeping with the published literature cell morphology of MDS-MSCs was similar to that of control MSCs and to the morphology reported in literature [1, 2, 31]. Furthermore, MSCs derived from MDS subtypes and controls were able to differentiate into osteogenic and adipogenic lineages. The results suggest that the MDS-MSCs are comparable to controls with respect to cell morphology and differentiation ability and are consistent with previous studies done in other populations [9, 12, 14, 15]. It was not possible to maintain two of the primary cultures (patient numbers 13-014 and 14-054) which were contaminated after 4-5 days and two other cultures (patient numbers 13-001 and 14-050, whose morphological assessment and differentiation studies were done) due to contaminations at passage 2 and excluded from further experiments.

The data available on functional properties of MDS derived MSCs are contradictory. Some researches have shown that the MSC layers derived from MDS patients are able to support the growth of mononuclear cells or hematopoietic progenitor cells at least for many weeks in culture [10, 12, 15]. Flores-Figueroa et al. in 2008 showed that MDS derived MSCs are able to sustain the growth and development of hematopoietic cells as efficiently as normal MSCs as evident by the production of myeloid cells throughout their culture period [15]. In contrast, some other researches have shown that MSCs of MDS exhibit defective hematopoietic supportive capacity compared to healthy MSCs [13, 37, 40]. Many studies have shown deficient growth characteristics in MDS derived MSCs compared to the healthy cells [14, 37, 40–42]. MSCs isolated from MDS patients have previously shown decreased growth and proliferative capacities with increased population doubling time and low proliferation rates with increased apoptosis [35–38]. In contrast, in our study the growth characteristics of MDS-MSCs were similar to that of control MSCs in terms of growth rates and PDTs. No significant difference was observed between the MDS-MSCs and control MSCs (*p* > 0.05). Further, the MSCs derived from all subtypes adhered to the flask between 15 and 48 hours after seeding P3 cells at log phase and showed exponential growth from day 2 to day 10. We did not observe significant changes in the* in vitro* growth characteristics of MSCs derived from MDS subtypes (RCUD, RCMD, RAEB, and del(5q)) in terms of PDTs (*p* > 0.05). Further, CFU-F frequencies of P2 MDS-MSCs were not significantly different from that of control MSCs (*p* > 0.05).

CD markers have shown to be important in hematological disorders [19]. Researches have reported the varied expression of surface CD markers of MSCs in disease states [19, 20]. Researches have identified the possible involvement of CD73 in MSC differentiation via A2AR (A_2A_ adenosine receptor) signaling [43]. CD105 which is a glycoprotein has a role in angiogenesis and has strong interactions with TGF-beta signaling and thus has a role in cancer development [44]. The expression patterns of these markers in disease conditions deserve attention due to their regulatory roles in cancer development and possible use in cancer therapy [45]. While some studies show that MDS-MSCs express normal levels of CD73, CD90, and CD105 [14, 15] some researchers have observed decreased levels of CD90 and CD105 expression in MDS-MSCs [19, 20]. Campioni et al. in 2006 reported that the cultured BM-MSCs of patients with hematological malignancies express CD90 and CD105 in lower frequencies compared to the normal BM-MSCs [19] and in another study they proposed that the low CD90 expression on BM-MSCs might be related to the deficiency in immunomodulatory properties of MSCs on T cell proliferation [20]. However in our study, the percentage of cells of P2 MDS-MSCs that express CD90 was very high (>95%) and was comparable to controls. Further, the mean fluorescent intensity for CD90 expressed by MDS-MSCs was also very high (4th logarithmic decade). RCMD-MSCs demonstrated low CD73 and high CD105 expression frequencies with variations in the expression levels (10^1^–10^4^ intensity units) compared to the control MSCs and MSCs of other MDS subtypes. The variations observed in expression pattern of positive markers may have a clinical significance in the pathogenesis of MDS. However, these findings need to be validated with higher patient numbers. The inconsistencies in MSC immunophenotypic profiles reported in literature may be due to differences in culture conditions including culture media used by different laboratories or may be due to the heterogeneity of MDS patients.

Identification of cytogenetic profiles of MSCs in MDS is important to determine the cause of functional differences in marrow stroma in MDS. Here we analyzed the karyotypes of culture-expanded MDS-MSCs. To optimize the karyotyping protocol for MSCs, we tested different colcemid exposure times: 2 hr, 5 hr, and overnight (12–14 hr). The overnight (12–14 hours) incubation with colcemid yielded the highest number of analyzable metaphase spreads and was considered as the optimum for MSCs in our laboratory conditions. This result also supports the findings by Muntión et al. in which they have found that 15 hour colcemid exposure time as the optimum for MSC karyotyping [46].

Detection of chromosomal abnormalities in MDS-MSCs in the absence of BM karyotypic abnormalities is an important finding in this study. These abnormalities were particularly observed in RCMD patients (60%, 3/5). Of MSCs studied, 31% (5/16) were cytogenetically abnormal and included structural and numerical abnormalities with loss of chromosomes. The percentage of presence of structural chromosomal aberrations in MDS derived MSCs is found to be low in some studies [21, 22], while there are other reports that claim for higher rates of having abnormal MSCs in MDS [12, 23]. In Blau et al. research in 2011 on MDS/AML patients it was found that the presence of genetic abnormalities was as low as 16% of the patients studied [21]. According to Kim et al. the detection of chromosomal aberrations in marrow stromal cells was only 5% (1/21) MDS patients studied [22]. In contrast Song et al. in 2012 have observed higher rates of cytogenetic aberrations (68% (15/22)) in MDS derived MSCs [23]. A study by Flores-Figueroa reported that 55.5% (5/9) of MDS-MSCs as having cytogenetic abnormalities [12]. In our patient group the detection of cytogenetic aberrations were moderate (31%) which is higher than some of the previous studies [21, 22] but lower than some other reports [12, 23].

The observed structural abnormalities included 46,XX,der(3) and 46,XY,del(7)(p21p22) in two RCMD patients and 46,XY,del(6)(q23q26) in a RAEB II patient. Only a very few studies describe the structural genetic aberrations of MSCs in relation to MDS. One study describing chromosomal abnormalities in MSCs by Blau et al. in 2007 reported structural abnormalities in MSCs including a derivative 7 and del(3)(p21) [9]. In another study same group had found del(7q) in their MDS/AML MSC populations [21]. Aberrations of chromosomes 3 and 7 in HSC compartment are found to be present in MDS [47]. Recent finding suggests that MDS patients with interchanged or inverted pieces chromosome 3 has higher risk of evolving into AML [47]. Deletion 6q results in loss of tumor suppressor genes and is commonly seen in lymphoid malignancies [47]. Here we report the presence of aberrant chromosome 3 and deletions in chromosomes 6 and 7 in MDS-MSCs which may have implications on the functioning of these cells in hematopoiesis and pathogenesis of MDS [9].

Previous studies report that the cytogenetic abnormalities are predominantly present in MSCs in those cases with chromosomal abnormalities in their hematopoietic counterpart [9, 12]. It has been also reported that the aberration rate was lower (33%) in MSCs of patients with normal karyotypes [22]. Interestingly, in this study we observed chromosomal aberrations in MSCs of MDS patients with normal BM karyotypes. Similarly, the three patients who had chromosomal abnormalities in their BM karyotypes did not show cytogenetic alterations in their MSC karyotypes. This includes the del(5q) patient whose MSCs exhibited a normal karyotype with FISH being negative for deletion 5q31.2/5q33. Hence we believe presence of chromosomal aberrations in MSCs could be regarded as an independent event and our findings may not support the idea that MSCs in MDS are derived from the same “neoplastic clone” even though such a possibility cannot be ruled out.

We also observed that most of the spreads showed randomly missing chromosomes and loss of chromosome material. This was particularly observed in three samples (RCUD, RCMD, and RAEB) where more than 90% of the spreads captured had missing chromosomes. Flores-Figueroa et al. and Blau et al. in their studies reported the presence of chromosomal aberrations in MDS derived MSCs involving loss of chromosomal material [9, 12]. Loss of chromosomal material could be evidence of genetic instability of MDS derived MSCs and may suggest the potential involvement in the pathogenesis of MDS.

It was reported that the MSCs cultured from the normal bone marrow show normal karyotypes after many passages [48]. However, we do not rule out the possibility of developing genomic changes under different culture conditions. Hence to avoid such possibilities, two independent cultures were set from passage 3 cells which contained more homogeneous cell population.

## 5. Conclusions

This study shows that MSC morphology, positivity to MSC CD markers, and differentiation ability remain unchanged in* de novo* MDS. MDS-MSCs demonstrated karyotypic abnormalities independent of BM karyotypes. RCMD patients showed higher degree of presence of abnormalities in their MSC populations with low CD73 and high CD105 expression frequencies compared to the MSCs of controls and other subtypes. Further, in contrast to some previous reports here we show that the CD90 expression on BM-MSCs of all subtypes is very high and is comparable to control-MSCs. Presence of distinct genetic abnormalities compared to their corresponding hematopoietic counterpart in MDS stromal cells supports the view that the occurrence of genetic abnormalities in MSC compartment in MDS could be an autonomous event from that of their hematopoietic counterparts. While this study supports and confirms previously published data in relation to the morphology, immunophenotype, and differentiation ability of MDS-MSCs, this study also emphasizes the importance of studying MDS subtypes separately as MDS subtype dependent variations were observed in CD marker expression levels and cytogenetic profiles. Even though variabilities of methodologies of isolation and expansion or culture conditions cannot be ruled out as causes for contradictory data from different MDS-MSC research groups, one of the main reasons as we believe is the heterogeneity of the MDS disease biology and the distinct pathophysiology of MDS subtypes. Therefore more and more data on MDS derived MSCs of different populations and data on MDS subtypes are required in order to clarify the existing controversies. Taken together, the study has implications on expanding our knowledge on morphological and clinical heterogeneity of MSCs in MDS.

## Supplementary Material

Supplementary figure 1and figure 2 show the CD marker expression plots of MSCs derived from representative MDS patients. The forward scatter versus side scatter dot plot shows the gate of the MSC population (R1). The MDS-MSCs were positive for CD73, CD90 and CD105 and were negative for CD34 and CD45 surface markers.
CFU-F assay results for MDS-MSCs and control-MSCs are shown in supplementary figure 3. Mean CFU-F frequency of MDS-MSCs was 19.63±4.91 (range: 7-41; n = 16) and that of controls was 17.93 ± 4.25 (range: 8-35; n = 5). CFU-F frequencies of MDS-MSCs were not significantly different from that of control MSCs (p > 0.05).

## Figures and Tables

**Table 1:** Cytogenetic findings of bone marrow and MSCs of MDS patients.

Patient ID	MDS subtype	Age	Sex	Cytogenetics of BM	Cytogenetics of MSCs
14-020	RCMD	68	F	46,XX	46,XX,der(3) [8]
14-058	RCUD	73	F	46,XX	46,XX
14-066	RCUD	31	F	Random loss of chromosomes	ND
14-069	RCUD	73	F	46,XX	46,XX
14-072	RAEB II	55	M	Failed	46,XY,del(6)(q23q26) [5]
14-079	RAEB II	75	M	Failed	46,XY
14-080	RCMD	74	M	46,XY	38~43,XY,del(7)(p21p22) [3]
14-081	Del(5q)	67	F	46,XX,del(5)(q12q34) [5], 46,XX.ish del(5)(q31.2q33)(CDC25C-,EGR1-,RPS14-)	46,XX FISH negative for del(5)(q31.2q33)
14-083	RCUD	54	F	46,XX	46,XX
14-086	RCMD	49	F	46,XX,del(12)(p12) [3]	46,XX
14-089	RCUD	70	M	Failed	31~46,XY
14-090	RCUD	59	F	46,XX,del(11)(q22q24) [7]	46,XX
15-001	RCMD	65	F	46,XX	46,XX
15-015	RAEB I	66	M	46,XY	46,XY
15-017	RCMD	50	M	46,XY,+19	33~46,XY
15-018	RCUD	62	M	46,XY	46,XY

RCUD: refractory cytopenia with unilineage dysplasia, RAEB: refractory anaemia with excess blasts, RCMD: refractory cytopenia with multilineage dysplasia, and ND: no analyzable metaphases obtained.
