# Supplementary material for: Phenotypic and Cytogenetic Characterization of Mesenchymal Stromal Cells in De Novo Myelodysplastic Syndromes
Source: Anal Cell Pathol (Amst). 2016 Aug 29;2016:8012716. doi: 10.1155/2016/8012716 (PMC5021885; doi:10.1155/2016/8012716)
Supplement: Supplementary file 1 — Supplementary figure 1and figure 2 show the CD marker expression plots of MSCs derived from representative MDS patients. The forward scatter versus side scatter dot plot shows the gate of the MSC population (R1). The MDS-MSCs were positive for CD73, CD90 and CD105 and were negative for CD34 and CD45 surface markers. CFU-F assay results for MDS-MSCs and control-MSCs are shown in supplementary figure 3. Mean CFU-F frequency of MDS-MSCs was 19.63±4.91 (range: 7-41; n = 16) and that of controls was 17.93 ± 4.25 (range: 8-35; n = 5). CFU-F frequencies of MDS-MSCs were not significantly different from that of control MSCs (p > 0.05). [file 8012716.f1.pdf]

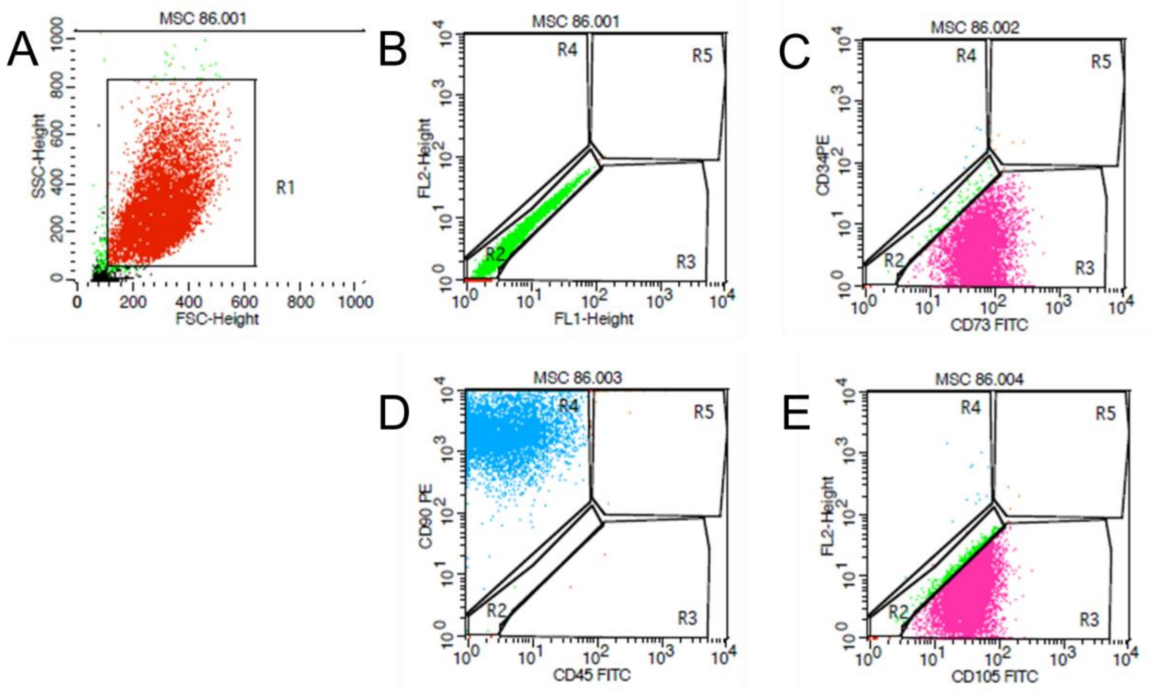

Supplementary Figure 1: Immunophenotypic characteristics of MSCs from a representative MDS patient. The forward scatter versus side scatter dot plot shows the gate of the MSC population (R1). The MDS-MSCs were positive for CD73(C-R3), CD90 (D-R4) and CD105 (E-R3) and were negative for CD34 (C-R4) and CD45 (D-R3) surface markers.

A

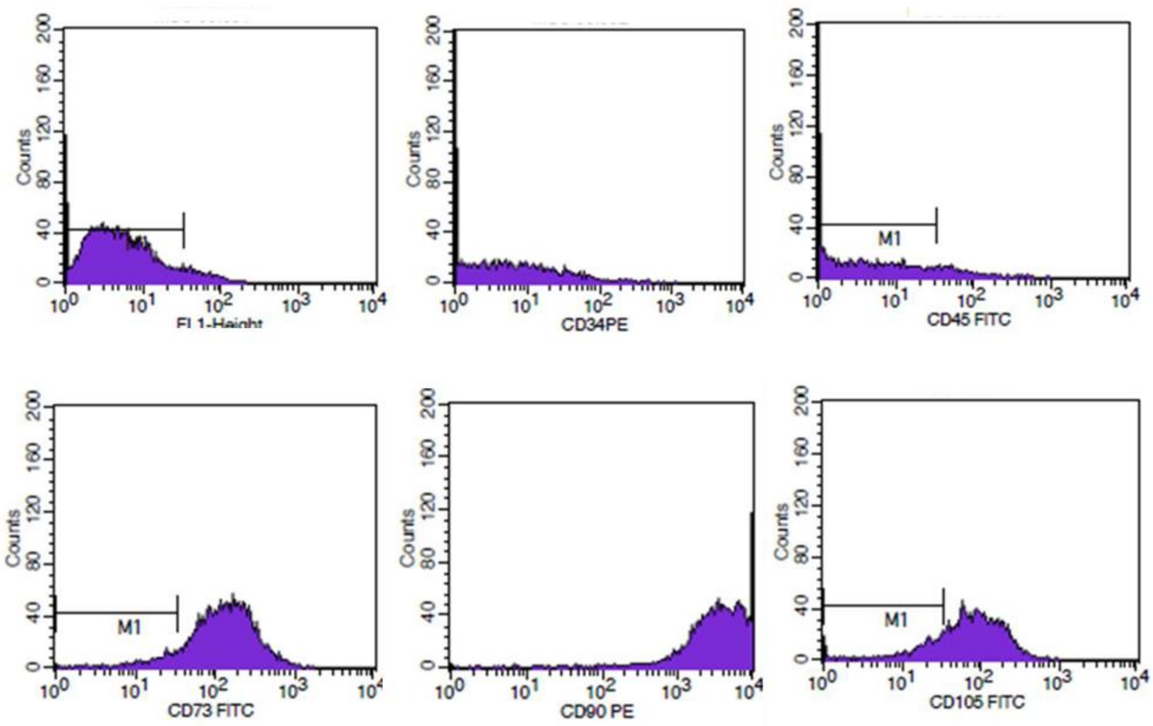
Counts
M1
FL1-Height
CD34PE
CD45 FITC
CD73 FITC
CD90 PE
CD105 FITC

B

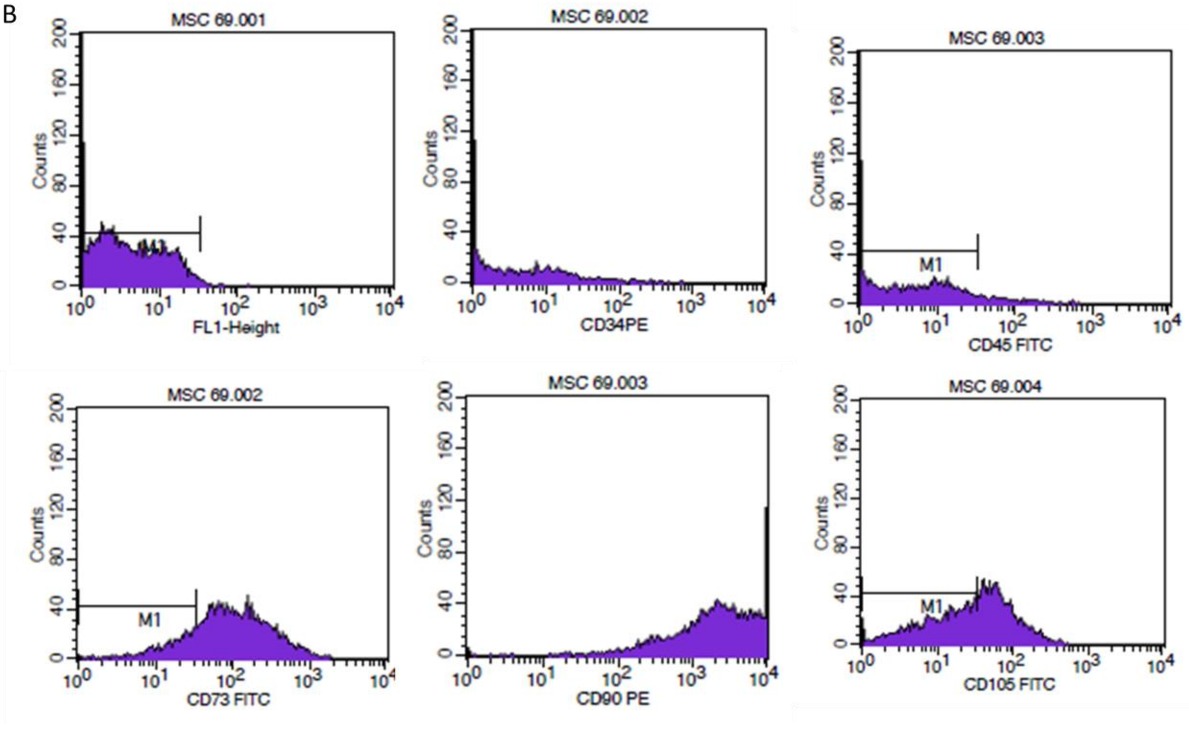
MSC 69.001
MSC 69.002
MSC 69.003
MSC 69.002
MSC 69.003
MSC 69.004
Counts
M1
FL1-Height
CD34PE
CD45 FITC
CD73 FITC
CD90 PE
CD105 FITC

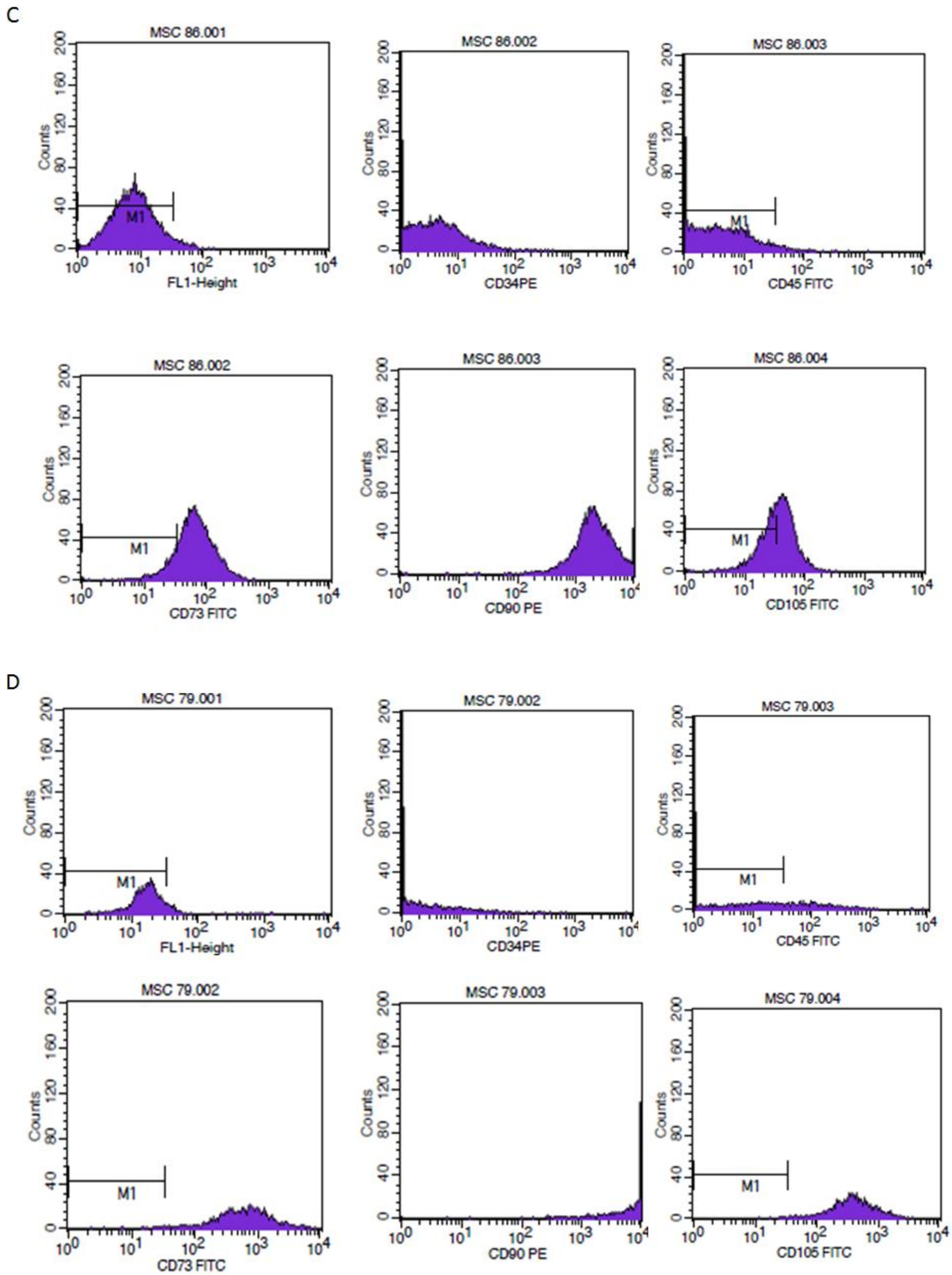

Supplementary Figure 2: Flowcytometric analysis of MSCs. Representative FACs plots of A-control MSCs, B-RCUD MSCs, C-RCMD MSCs and D-RAEB MSCs expressing CD markers CD34, C45, CD73, CD90 and CD105.

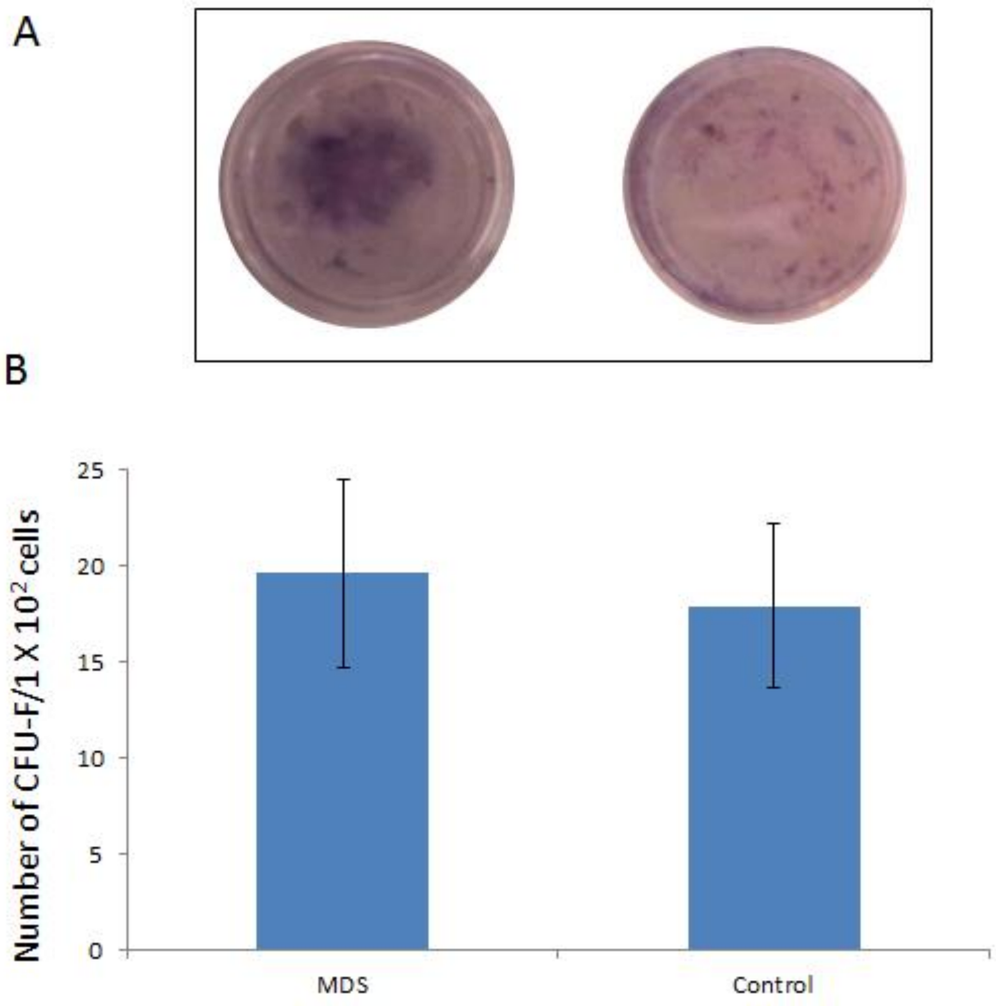

Supplementary Figure 3: A- CFU-F assay for MDS-MSCS and control-MSCs. B-Mean CFU-F frequency of MDS-MSCs was 19.63±4.91 (range: 7-41; n = 16) and that of controls was 17.93±4.25 (range: 8-35; n = 5). CFU-F frequencies of MDS-MSCs were not significantly different from that of control MSCs (p>0.05).
